# Emerging role of nuclear factor erythroid 2-related factor 2 in the mechanism of action and resistance to anticancer therapies

**DOI:** 10.20517/cdr.2019.57

**Published:** 2019-09-19

**Authors:** Poornima Paramasivan, Ibrahim H. Kankia, Simon P. Langdon, Yusuf Y. Deeni

**Affiliations:** ^1^Division of Science, School of Applied Sciences, Abertay University, Dundee DD1 1HG, United Kingdom.; ^2^Department of Biochemistry, Faculty of Natural and Applied Sciences, Umaru Musa Yar’adua University, Katsina PMB 2218, Nigeria.; ^3^Cancer Research UK Edinburgh Centre and Edinburgh Pathology, Institute of Genetics and Molecular Medicine, University of Edinburgh, Crewe Road South, Edinburgh EH4 2XU, United Kingdom.

**Keywords:** Cancer, nuclear factor E2-related factor 2, drug resistance, receptor tyrosine kinase inhibitor, DNA damage and repair response, targeted therapy, anticancer therapeutics

## Abstract

Nuclear factor E2-related factor 2 (NRF2), a transcription factor, is a master regulator of an array of genes related to oxidative and electrophilic stress that promote and maintain redox homeostasis. NRF2 function is well studied in *in vitro*, animal and general physiology models. However, emerging data has uncovered novel functionality of this transcription factor in human diseases such as cancer, autism, anxiety disorders and diabetes. A key finding in these emerging roles has been its constitutive upregulation in multiple cancers promoting pro-survival phenotypes. The survivability pathways in these studies were mostly explained by classical NRF2 activation involving KEAP-1 relief and transcriptional induction of reactive oxygen species (ROS) neutralizing and cytoprotective drug-metabolizing enzymes (phase I, II, III and 0). Further, NRF2 status and activation is associated with lowered cancer therapeutic efficacy and the eventual emergence of therapeutic resistance. Interestingly, we and others have provided further evidence of direct NRF2 regulation of anticancer drug targets like receptor tyrosine kinases and DNA damage and repair proteins and kinases with implications for therapy outcome. This novel finding demonstrates a renewed role of NRF2 as a key modulatory factor informing anticancer therapeutic outcomes, which extends beyond its described classical role as a ROS regulator. This review will provide a knowledge base for these emerging roles of NRF2 in anticancer therapies involving feedback and feed forward models and will consolidate and present such findings in a systematic manner. This places NRF2 as a key determinant of action, effectiveness and resistance to anticancer therapy.

## Introduction

### Cancer drug resistance

A major road block in cancer patient care is the development of resistance where cancer cells become tolerant to pharmaceutical treatments^[1]^. In clinical practice, low therapeutic index and dose limited toxicity are crucial problems associated with drug resistance. Despite encouraging progress in drug discovery and enhanced understanding of the molecular mechanisms of drug action, many cancer patients still succumb to drug resistance. Even with the introduction of new “targeted” drugs, drug resistance remains the foremost concern in cancer treatment with reports suggesting that the resistance mechanisms to these agents are frequently similar or identical to those of classical chemotherapeutic agents. Cytoprotective mechanisms against therapeutic/cytotoxic compounds which evolve in mammals continue to be daunting challenges for successful treatment of cancer^[2]^. Diverse and complex biochemical and genetic mechanisms underlie the drug resistance phenomenon^[3]^.

### Mechanisms of drug resistance

**Figure 1:**
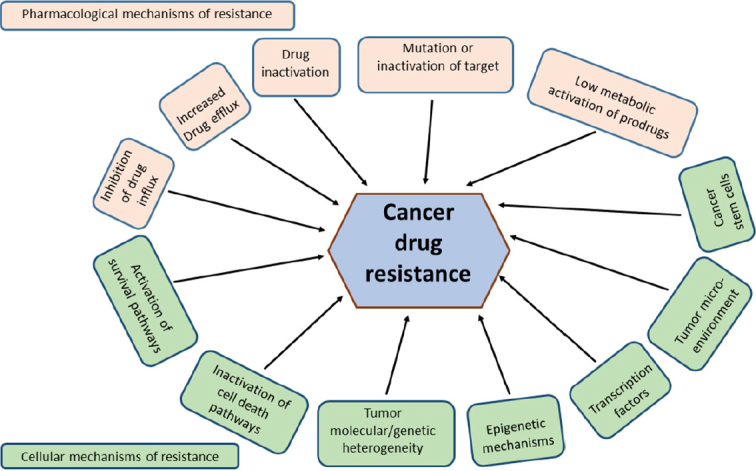
Plausible mechanisms of cancer drug resistance. The mechanisms can generally be classified as pharmacological and cellular physiological mechanisms, which either feed into or feed out of the well-known and characterised hallmarks of cancer^[12]^

Mechanistically, resistance phenomena may frequently be explained by mutation or over expression of drug target proteins and/or inactivation of drugs by a reduction in uptake or enhanced detoxification and removal of drugs^[4-6]^. With a highly adaptable nature, cancer cells become resistant through the activation of survival pathways and the inactivation of downstream death signalling pathways. The influences of epigenetics, tumor microenvironment and cancer stem cells along with molecular and genetic heterogeneity of tumors have also been implicated in the development of drug resistance^[7-10]^ [Figure 1]. Rigorous research has dramatically increased our knowledge about cancer drug resistance associated genes, proteins and their mechanisms of action. Studies on the control of cellular gene expression programs have highly influenced the understanding of genetic alterations in disease. Importantly, deregulation of transcription factors (TFs) was found to be a pervasive phenomenon in the pathogenesis of many forms of cancer^[11]^.

### TFs and drug resistance

Over 2000 different TFs are encoded by the human genome. TFs are expressed in a cell type specific manner and regulate an array of cellular processes by coordinating their gene expression programs. The contribution of mutations in the TFs in tumorigenesis have been known for decades and studies have demonstrated that the autoregulatory circuitry of the cell can be altered by the overexpressed TFs^[11]^. Various proto-oncogenes and tumor suppressor genes encode transcription related factors that alter the drug sensitivity of cells^[12]^. Drug induced responses can be affected by TFs and TFs can induce transient or acquired drug resistance^[13]^. Many of the TFs including nuclear factor E2-related factor 2 (NRF2) have been demonstrated to be overexpressed in drug resistant cancers [Table 1]. It is of interest that most of these TFs highlighted in Table 1 are directly or indirectly regulated or influenced by cellular NRF2 status and/or function. In this review, we discuss the role of NRF2 in the mechanism of action and resistance to anticancer therapies.

**Table 1:** Some TFs that contribute to drug resistance

Transcription factors	Cancer type	Drug resistance	Reference
SP1	Ovarian cancer, Leukemia	Cisplatin, Doxorubicin	[14,15]
YB1	Gastric cancer	Cisplatin	[16]
NF-κB	Ovarian cancer	Cisplatin	[17]
NRF2	Breast cancer, Non-small cell lung cancer, Ovarian Cancer, Endometrial cancer, Gallbladder cancer, Pancreatic cancer, Renal cancer	Mitoxanthrone, Doxorubicin, Cisplatin, Oxaliplatin, Gemcetabine, Temsirolimus, Gefitinib, Erlotinib, Lapatinib, Imatinib, Afatinib, Axitinib, Sunitinib, Osimertinib, Trastuzumab,Pertuzumab	[18-34]
YAP	Hepatocellular carcinoma	Doxorubicin	[35]
HIF1-α	Gastric cancer	5-fluorouracil	[36]
c-MYC	Non-small cell lung cancer	Gefitinib, Erlotinib	[37]
ATF2	Pancreatic cancer	Gemcitabine	[38]
ZNF143	Epidermoid cancer, Prostate cancer	Cisplatin	[39]

TFs: transcription factors

## NRF2 and its function

NRF2 is a member of the bZIP family of TFs^[40]^. While the basic region, just upstream of the leucine zipper region is responsible for DNA binding the acidic region is required for transcriptional activation. In mammals, the CNC (Cap “N” Collar) family is composed of four closely related proteins; p45-NF-E2^[41]^, NRF1^[42]^, NRF2^[43,44]^ and NRF3^[45]^. Others are two remotely related proteins; BTB and CNC homology 1 (BACH1)^[46]^ and BTB and CNC homology 2^[47]^. The roles of some of these mammalian CNC factors have been extensively studied. These proteins, form heterodimers with other b-ZIP proteins, such as small musculoaponeurotic fibrosarcoma K, G and F (MafK, MafG, MafF), to function as TFs^[48]^. For example, the pattern of heterodimeric association between NRF2 and small Mafs, is that the small Maf protein provides DNA binding activity to NRF2, while NRF2 activates transcription via its transactivation domain^[49]^. Hence, NRF2 cannot bind to the ARE as a monomer, but requires dimerization with one of the small Maf proteins in order to bring about transactivation^[50]^.

**Figure 2:**
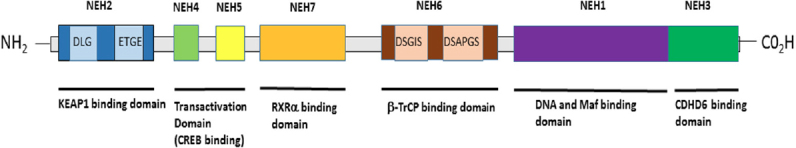
Structural and functional domains of NRF2. A Schematic representation of the human/mammalian NRF2 structure and function domains. There are 7 highly conserved regions in NRF2 that are referred to as NEH domains. From the N-terminal to the C-terminal of NRF2, the NEH2 domain contains the DLG/ETGE motifs that facilitate NRF2 interaction with KEAP1 and for KEAP1-dependent NRF2 proteasomal degradation. The NEH2 domain also contain a lysine residues rich site that is directly ubiquitylated by the Cul3/Rbx1/E3 cullin-based E3 ubiquitin ligase substrate adaptor complex, as well as a first NLS sequence between the amino acids 42 and 53. The NEH4-5 domains facilitate the interaction of NRF2 with Hdr1 and other proteins like p300 and CBP to activate NRF2-dependent transcription; also, a NES is located between amino acids 191-202 in the NEH5 region. The NEH7 domain contains sites for interaction with the RARs (RXR-α and RAR-α) that facilitates NRF2 transcriptional repression. The NEH6 domain contains two specific sites of interaction with the β-transducing repeat-containing protein (βTrCP ubiquitin ligase; the binding by βTrCP to the DSGIS motif requires the prior phosphorylation of NRF2 in Ser344 and Ser347 by Gsk-3β, but the interaction of βTrCP with the DSPAGS motif of NRF2 is direct. The association of NRF2 with βTrCP leads to Cul1-mediated ubiquitination, followed by NRF2 proteasome degradation. The NEH1 domain contains the CNC bZIP region, which is required for DNA binding and dimerization with small Maf proteins and other TFs; also, there is a second NES sequence between amino acids 553 and 562. Finally, the NEH3 region is another transactivation domain that contains a second NLS sequence between amino acids 595 and 601. NRF2: nuclear factor E2-related factor 2; NLS: nuclear localisation signal; NES: nuclear export signal; CNC bZIP: Cap'n'Collar basic leucine zipper; TFs: transcription factors; Maf: musculoaponeurotic fibrosarcoma

NRF2 contains seven basic domains, namely Neh1-Neh7. The Neh1 domain has been shown to bind to ubiquitin-conjugating enzymes to enhance the stability and the transcriptional activity of NRF2. The second domain, known as Neh2 [Figure 2], possesses two essential motifs known as DLG, which has less affinity for Kelch-like ECH-associated protein 1 (KEAP1), and ETGE, which has a high affinity for the interaction between NRF2 and KEAP1^[51,52]^. The Neh3 domain contains a carboxy-terminal which associates with transcription co-activators such as chromodomain helicase DNA binding protein 6, which is responsible for the transactivation of ARE-dependent genes. Both Neh4 and Neh5 domains bind with cAMP response element binding protein, which facilitates the transactivation of NRF2 target genes. These two transactivation domains are also reported to interact with the nuclear cofactor known as receptor-associated coactivator 3/amplified in breast 1/steroid receptor coactivator-3 (SRC-3), thereby leading to an improved NRF2-ARE gene expression. The Neh5 domain also possesses a redox-sensitive nuclear export signal that mediates the cellular localisation of NRF2. The sixth domain, known as Neh6, contains a domain that is rich in serine amino acids, and contains two motifs known as DSGIS and DSAPGS. The Neh6 domain is involved in the degradation of NRF2 even in stressed cells, where the half-life of NRF2 protein is longer than in unstressed conditions. The Neh6 domain also offers stability control of NRF2 when NRF2 is in the NRF2-KEAP1 complex^[53-55]^. The Neh7 domain is a recent discovery and has been found to specifically interact with RXRα, a nuclear receptor that inhibits the NRF2-ARE signalling pathway^[51,56-60]^.

## NRF2 signalling pathway and regulation

**Figure 3:**
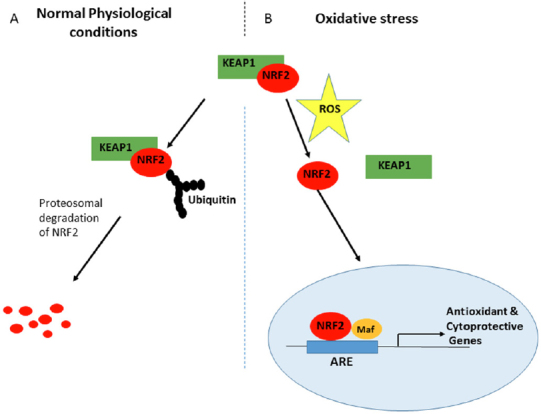
Redox regulation of NRF2-KEAP1 signalling. A: Under normal physiological (homeostatic) conditions, KEAP1 interacts with NRF2 in the cytosol, promoting its polyubiquitylation and subsequent proteasomal degradation by the substrate adaptor for cullin-based E3 ubiquitin ligase complex, resulting in little NRF2 that is sufficient for the maintenance of cellular homeostasis or absence of NRF2 transactivation; B: In contrast, under oxidative stress conditions, the binding of KEAP1 to NRF2 is greatly impaired to compromise the likelihood of NRF2 ubiquitylation. Consequentl, a greater fraction of NRF2 molecules in the cytosolic pool can translocate into the nucleus, wherein NRF2 interacts with sMAF proteins, then binds to DNA and other transcription partners to form a heterodimeric nuclear complex, which induces the transcription of several antioxidant and cytoprotective genes. NRF: nuclear factor E2-related factor 2; sMAF: small musculoaponeurotic fibrosarcoma

NRF2 is maintained at low concentration in the cytoplasm under normal basal conditions, due to control by KEAP1 that targets and presents NRF2 for ubiquitination and subsequent proteasomal degradation^[53,54,56]^. However, since degradation of NRF2 by the 26S proteasome requires prior ubiquitination of the substrate molecule, recognition and targeting of the NRF2 protein by the ubiquitin ligases may represent a critical rate-limiting step. NRF2 activation has been found to be promoted by oxidative stress in the cells. An increase in the level of NRF2 in response to stress leads to its dissociation from KEAP1, and is mediated by a post-transcriptional mechanism rather than an increase in NRF2 mRNA levels. Hence activation of NRF2 has an important role in its stability and transcriptional activity^[61-63]^. The KEAP1-NRF2 complex is formed in the cytoplasm, where NRF2 is ubiquitinated and degraded in the event of normal basal conditions. In the event of stress, NRF2 dissociates from KEAP1 and translocates into the nucleus [Figure 3] where it heterodimerises with sMAF and then binds to ARE for initiation of expression of cytoprotective and detoxifying genes^[54]^.

Studies have shown that the process where KEAP1 interacts with NRF2 is through a mechanism called “hinge and latch” in which two motifs of NRF2 (DLG and ETGE) bind with the KEAP1 homodimer. The ETGE motif possesses a higher affinity for KEAP1 than the DLG motif and acts as a hinge, whilst DLG acts as a latch^[51,64]^. NRF2 first binds with KEAP1 at the ETGE site where there is high affinity through the hinge, then at the DLG site by the latch. Under normal basal conditions, NRF2 remains attached to KEAP1 through the hinge and latch interaction until activated by its inducers throwing it into an oxidative stress state, when it then dissociates from KEAP1 in the cytoplasm. This free NRF2 then translocates to the nucleus, where it binds with sMAF proteins to form a heterodimer, and then transactivates ARE-driven gene expression that leads to the expression of many cytoprotective and detoxifying genes^[51,55,65]^. The phosphorylation of NRF2 by a series of protein kinases is reported to result in changes in the NRF2-KEAP1 complex and subsequent stabilisation of NRF2, which promotes the dissociation of NRF2 from KEAP1 and its accumulation in the nucleus^[53,54]^.

NRF2 activation involves two basic pathways: canonical and non-canonical. The canonical pathway accounts for the primary mechanism of NRF2 activation. This is based on the dissociation of NRF2 from KEAP1 in the cytoplasm leading to the translocation of NRF2 into the nucleus where it dimerizes with sMAF proteins, and then binds to ARE-carrying promoters to subsequently initiate the gene expression of cytoprotective and detoxifying enzymes^[60,66]^. The activation of the phosphoinositide 3-kinases (PI3K)/Akt signalling pathway and stresses on the endoplasmic reticulum are some of the mechanisms that can lead to nuclear accumulation of NRF2 and increased ARE-driven gene expression^[60,67,68]^. The non-canonical pathways of NRF2 activation involve numerous proteins with motifs similar to the ETGE motif in NRF2 competing with NRF2 for KEAP1 binding. In this process, NRF2 loses out in the binding to KEAP1 and therefore becomes free, leading to its accumulation in the cytoplasm^[60]^. This is a harbinger to the eventual ease of activation and translocation of NRF2 to the nucleus. Proteins that compete with NRF2 include p62, a protein that is known to contain the STGE motif, dipeptidyl peptidase 3 and a partner and localiser of BRCA2^[60,66]^.

NRF2 has also been described as “a guardian of healthspan and gatekeeper of species longevity”^[69]^. It has been associated with regulating aging and can be up-regulated by longevity promoting interventions including dietary and pharmacological approaches^[70]^. With aging, NRF2 activity is diminished in many cell types with decline of oxidant stress resistance^[70]^. Conversely, strategies that enhance NRF2 activity such as mutations that increase NRF2 nuclear activity can increase mouse lifespan^[71]^ while loss-of-function mutations in *Keap1* can extend *Drosophila* life span^[72]^. Positive lifestyle influences that include calorific restriction and exercise have been associated with both enhanced NRF2 activity and increased longevity^[70]^.

Early NRF2 research pinpointed the role of NRF2 in preventing cancers^[73]^. For instance, a study by Ramos-Gomez *et al*.^[74]^ reported that NRF2 null mice are more susceptible to carcinogen-induced tumors than their NRF2 wild-type counterparts. Moreover, another study by Pearson *et al*.^[75]^ compared the effects of caloric restriction on NRF2 wild-type and NRF2 null mice, and implicated NRF2 as playing an important role in preventing cancer in the caloric restricted mice.

Studies by Padmanabhan *et al*.^[76]^ and Singh *et al*.^[77]^ reported on activation of NRF2 in cancer, describing mutations and polymorphisms in KEAP1 in lung cancer tissues and cell lines. Increased NRF2 activity in cancer was reported to have a role in increased cancer cell survival and resistance to chemo- and radiotherapy, which could lead to poor prognosis^[78]^. Identified differences in the clinical manifestation of tumors suggest that those with sustained NRF2 activation are distinct from those without^[73]^. An elegant study has demonstrated that dysregulation of the NRF2/KEAP1 system can impact lung cancer survival by increasing the metastatic potential of lung cancer cells^[79]^. Approximately 30% of non-small lung cancers have mutations in either *KEAP1* or in *NFE2F2* resulting in stabilisation of NRF2. This study demonstrated that NRF2 activation can lead to a metastatic programme by inhibiting the heme and FBXO22-mediated degradation of BACH1. In turn, this suggests that Heme oxygenase inhibitors could represent a potential therapeutic strategy. Following the findings of the role of NRF2 in chemoresistance, researchers have focussed on identifying NRF2 inhibitors to modulate NRF2 to overcome chemoresistance^[51,55,80-94]^.

## Dual roles of NRF2 - chemopreventive and chemoprotective

A number of studies have reported on the double-edged role of NRF2. It displays a vital chemopreventive role in helping normal cells to tolerate stress, yet on the other hand it plays a crucial chemoprotective role in promoting carcinogenesis, drug resistance and cancer protection^[95-100]^. The chemopreventive role of NRF2 has been described in many studies^[101-104]^. The activation of NRF2 as a chemopreventive measure is an adaptive response to environmental and endogenous stresses that serves to render organisms resistant to chemical carcinogenesis and other forms of toxicity^[105-108]^. A wide variety of studies have reported several natural and synthetic compounds such as curcumin, xanthohumol, sulforaphane and oltipraz as inducing NRF2, which in turn leads to chemoprevention in cancers^[95,109-115]^. Alongside these phytochemicals, trace minerals including zinc and selenium, are essential to optimize NRF2-mediated resilience to oxidative stress^[116]^. NRF2 is known to be activated by a component of the gut microbiome, namely *Lactobacilli*^[117]^. Cellular reactive oxygen species (ROS) enzymatically is generated in response to contact with *Lactobacilli* in both mice and Drosophila and has effects against exogenous insults to the intestinal epithelium via the activation of NRF2 driven cytoprotective genes^[117]^. A new developing research field that seeks to link the microbiome, diet and lifestyle to molecular pathologies is molecular pathological epidemiology^[118,119]^. Since all of these factors influence NRF2 activation, it will be important to understand their combined interactions to assess their overall impact on NRF2 action. The ultimate goal of this would be to help predict impacts within individuals and eventually suggest recommendations for precision medicine.

In contrast, a number of studies have reported on the protective role of NRF2 in cancer leading to increased cancer cell proliferation and survival, a situation that may lead to drug resistance. One mechanism of NRF2 activation is a loss of interaction of the KEAP1 protein, leading to increasing and persistent nuclear accumulation of NRF2. This thereby activates antioxidant and anti-apoptotic gene expression, which in turn leads to drug resistance^[58,120-124]^. Interestingly some studies have reported on ways of overcoming this problematic side of NRF2. For example, transient transfection of NRF2-siRNA, sensitizes cancer cells to be more susceptible to Cisplatin and Doxorubicin^[122]^. In addition, the pharmacological inhibition of NRF2 as a way of overcoming chemoresistance and increasing the killing effect of anti-cancer drugs has been demonstrated^[84,125]^. Therefore, the pharmacological inhibition or genetic knockdown of NRF2 in cancer would help in overcoming chemoresistance^[53,54,82,84]^.

## NRF2 dysregulation and drug resistance - multiple mechanisms

NRF2 works as a double edge sword by regulating cellular antioxidants and providing survival advantages whereas the over-expression of NRF2 diminishes the toxic effect of anticancer agents. Recent studies have shown that constitutive high level expression of NRF2 leads to tumor formation and drug resistance in cancer cells. Somatic mutation in KEAP1/NRF2 is the foremost reason for NRF2 constitutive hyper activation. These mutations are often found in the regions of protein-protein interaction and compromise the KEAP1 checkpoint over NRF2^[126]^. Other reasons for NRF2 over-expression include epigenetic and post-translational modification, increased disruptor proteins, proto-oncogenes *etc*.^[127-130]^. In addition, polymorphisms in NRF2 lead to poor prognosis in lung and breast cancers, *etc*.^[131-133]^. Overexpression of Inhibitor of Apoptosis Stimulating Protein of p53 (iASPP), also known as Rel A-associated inhibitor, in tumor cells has been demonstrated to promote cancer growth and drug resistance through high NRF2 levels. Originally identified as a binding partner and trans-activity inhibitor of NF-kB/p65, iASPP has been shown to interact with Keap1. Thus, high levels of iASPP in tumor cells leads to insufficient binding of free Keap1 thereby freeing NRF2 to enter the nucleus^[134]^. Other proteins such as p21 and p62 can also directly bind to NRF2 or KEAP1 and disrupt the NRF2-KEAP1 interaction and NRF2 activation^[135,136]^.

When cancer cells acquire drug resistance during anticancer drug treatment, this process can be accompanied with higher NRF2 level in various cancer models. Cancer cells resistant to various anticancer drugs such as Tamoxifen, Oxaliplatin, Cisplatin, Doxorubicin, Etoposide, Imatinib, *etc*., have been reported to induce drug resistance by activating the NRF2 signalling pathway^[23-25]^. Recently it has been demonstrated that overexpression of NRF2 and its target genes in a Gefitinib-resistant non-small cell lung cancer cell line can be attributed to an acquired *Keap1* mutation. Furthermore, these Gefitinib resistant cells acquired cross-resistance to the irreversible EGFR-TKIs, Afatinib and Osimertinib^[28]^. Vorinostat is an effective histone deacetylase (HDAC) inhibitor and enhances the resistance of leukemia cells by promoting NRF2 nuclear translocation^[137]^.

Rigorous research has revealed that NRF2 elicits drug resistance in cancer cells via multiple mechanisms. NRF2 controls the expression of phase I and phase II drug metabolizing enzymes, phase III drug efflux transporters and other cytoprotective genes^[35]^. Table 2 summarizes several NRF2 controlled proteins that mediate drug resistance.

**Table 2:** NRF2 controlled proteins that mediate drug resistance

Proteins regulated by NRF2	Mechanism involved	Detoxification or transport phase
NQO1	Drug metabolism	I
CYP1B1	Drug metabolism	I
CBR1	Drug metabolism	I
AKR1B1	Drug metabolism	I
AKR1C1	Drug metabolism	I
GSTA1	Drug metabolism	II
GSTM1	Drug metabolism	II
GSTP1	Drug metabolism	II
UGT1A1	Drug metabolism	II
UGT2B7	Drug metabolism	II
MRP1	Drug efflux	III
MRP2	Drug efflux	III
MRP3	Drug efflux	III
MRP4	Drug efflux	III
MRP5	Drug efflux	III
BCRP	Drug efflux	III
SLC7A11	Drug influx	0
SLC3A2	Drug influx	0
SLC16A6	Drug influx	0

NRF2: nuclear factor E2-related factor 2; MRP1: mutidrug resistance protein 1; BCRP: breast cancer resistance protein; SLC7A11: solute carrier 7A11; SLC3A2: solute carrier 3A2; SLC16A6: solute carrier 16A6; UGT2B7: UDP glucuronosyltransferase 2 family, polypeptide B7; UGT1A1: UDP glucuronosyltransferase 1 family, polypeptide A1; GSTP1: glutathione S-transferase class Pi 1; GSTM1: glutathione S-transferase class Mu 1; GSTA1: glutathione S-transferase class alpha 1; AKR1C1: aldo-keto reductase family 1, member C1; AKR1B1: aldo-keto reductase family 1, member B1; CBR1: carbonyl reductase 1; CYP1B1: cytochrome P450, family 1, subfamily B, polypeptide1; NQO1: NAD(P)H: quinone oxidoreductase 1

### Phase I drug metabolizing enzymes

NRF2 regulates various phase I drug metabolizing enzymes which are reported to be overexpressed in tumors. A target of NRF2, NAD(P)H: quinone oxidoreductase 1 (NQO1) apart from catalysing the biotransformation of quinones, also acts as a superoxide scavenger to defend oxidative stress^[138,139]^. Overexpression of NQO1 in drug resistant breast, lung, colon and pancreatic tumors has been reported^[23,140-143]^. Even though it is widely accepted that NQO1 metabolizes and decreases anticancer drug toxicity, it also functions to increase the bioavailability of quinone containing alkylating agents such as Mitomycin C which is used to treat breast, lung, bladder and liver cancers^[144]^.

Cytochrome P450, family 1, subfamily B, polypeptide1 (CYP1B1), another NRF2 controlled enzyme metabolizes chemotherapeutic drugs such as Cyclophosphamide and Taxanes through hydroxylation. Upregulation of CYP1B1 results in altered structure of the drug and leads to cancer cell resistance towards anticancer agents such as Docetaxel, Paclitaxel, Flutamide^[145-148]^. Furthermore, the NRF2 dependent increase in carbonyl reductases leads to Doxorubicin resistance by reduction of the drug in leukemia and gastric cancer^[149,150]^; microsomal epoxide hydrolase acts through hydrolysis and is found to be overexpressed in gemcitabine resistant lung cancer cells^[151]^; Aldo-keto reductases lead to the resistance of lung cancer cells to Daunorubicin and Lidarubicin^[152]^.

### Phase II drug metabolizing enzymes

The contributions of NRF2 controlled Phase II metabolic enzymes such as Glutathione S-transferases (GST) and UDP-glucuronosyltransferases (UGT) were also reported in cancer drug resistance. GSTs catalyse the binding of electrophilic group of substrates to the sulfydryl on glutathione (GSH). This enables the following detoxification process of the compounds coordinated with multidrug resistance proteins^[153]^. Colorectal cancer patients with GSTP1 were found to show less positive responses than the GSTP1 deficient patients when treated with 5-fluorouracil (5-FU) or Oxaliplatin highlighting the role played by GSTP1 in drug resistance^[154]^. UGT1A4 was found to be a mechanism of intrinsic tamoxifen resistance by increasing the glucuronidation levels of active hydroxylated tamoxifen metabolites in patients with estrogen positive breast cancer^[155]^. In addition, microsomal glutathione transferase 1 was reported to be involved in Doxorubicin resistance in Ewing sarcoma^[156]^.

### Phase III Drug transport proteins

One of the most significant mechanisms of drug resistance is the overexpression of ATP-binding cassette (ABC) transporter super families commonly known as drug efflux pumps. These transporters utilize ATP and efflux/eliminate either cytotoxic drugs or targeted anticancer agents, thus decreasing the intracellular drug concentration and impair their efficacy. Thus, it is well known that the overexpression of drug transport proteins results in a resistant phenotype. The predominantly reported ABC transporters that contribute to drug resistance include P-glycoprotein/ABCB1, multidrug resistance-associated protein (MRP/ABCC) 1/2/3/4/5, and breast cancer resistance protein (BCRP/ABCG2). NRF2 positively regulates all these ABC family transporters conferring drug resistance in cancer cells. The ABC transport family contain ARE in their regulatory regions and their regulatory mechanism has been shown to be elicited through NRF2 mediated ARE - driven transcription^[138,157-160]^.

Abnormal expression of MRP1 increases the efflux of Doxorubicin reducing its cytotoxic potential in ovarian cancer, leukemia and non-small cell lung cancer cells^[160-162]^. NRF2 dependant MRP2 upregulation has been reported to be a cause of resistance towards platinum based therapy in small cell lung cancer and ovarian cancer^[163,164]^; Tamoxifen therapy towards breast cancer cells^[165]^. MRP3 was found to be more highly expressed in NSCLC than in SCLC, and NRF2 dependent MRP3 over expression causes intrinsic resistance of NSCLCs to anticancer drugs like vincristine, etoposide and Cisplatin^[162]^. MRP4 overexpression was found be one of the causes of Cisplatin resistance in gastric cancer cells^[166]^; NRF2 dependant MRP 5 upregulation contributes to Doxorubicin resistance in hepatocarcinoma^[93,167,168]^. BCRP has been reported to induce drug resistant phenotype of NRF2 dependance by increasing the efflux of 5-FU in breast cancer cells^[33]^ and Irinotecan, Topotecan and Mitoxantrone in colorectal cancer cells^[32,34]^.

### Phase 0 solute carrier transporters

Solute carrier (SLC) transporters which function mainly as influx transporters of hydrophilic drugs are another group of membrane transporters involved in drug resistance. SLC transporters could be beneficial for delivery drugs to cancer cells. Unfortunately, often the drug influx transporters are downregulated in drug resistant cancer cells. Nearly 30 SLC transporters are found to be involved in chemoresistance and several of them were identified as being controlled by NRF2^[169-171]^. Elaborative gene microarray studies of drug (Methotrexate, Cisplatin, Doxorubicin, Vincristine, Topotecan and Paclitaxel) resistant ovarian cancer cells has identified dysregulation of several NRF2 regulated SLC transporter genes after resistance development. These include SLC7A11 downregulation in Topotecan and Paclitaxel resistant cell line; SLC16A6 downregulation in paclitaxel resistant cell line with an upregulation of the same in Cisplatin, Doxorubicin, Methotrexate and Vincristine resistant cell line; SLC3A2 was upregulated in Methotrexate and Vincristine resistant cell line^[172]^. Another report suggests that the upregulation of SLC3A2 and SLCA11 are important in maintaining high levels of GSH contributing to Cisplatin resistance ovarian cancer cells^[173]^.

### Other NRF2 regulated genes/proteins

Overexpression of HO-1 has been observed in various drug resistant cancers such as breast cancer, gastric cancer, lung cancer and myeloid leukemia^[140,174-176]^. NRF2-induced GCLM expression up-regulates GSH synthesis and associated Imatinib resistance in chronic leukaemia cells has been reported^[24,177]^. The enzymes involved in the pentose phosphate pathway (PPP) including glucose-6-phosphate dehydrogenase, isocitrate dehydrogenase 1, malic enzyme 1, transketolase isoform 1 and transaldolase 1 were found to be regulated by NRF2^[178-181]^. Anticancer drug induced NRF2 hyperactivation transcriptionally activates these genes and facilitates PPP. This metabolic network adaptions helps in compensating drug induced metabolic block leading to apoptosis resistance^[35]^. Histone acetyltransferases-hMOF can physically interact with NRF2 and acetylates NRF2 at Lys588 which supports the maintenance of nuclear NRF2 leading to resistance of lung cancer cells towards Cisplatin, 5-FU and Bleomycin^[182]^. MicroRNA (miRNA), small non-coding RNA sequences that post-transcriptionally regulate mRNA sequences are also reported to be regulated by NRF2 and their contribution in drug resistance has also been noted [Table 3]^[183]^. For instance, NRF2 downregulation of miR200c and chemo resistance due to downregulation of miR200c have been reported in breast, ovarian and skin cancers^[184-186]^.

**Table 3:** miRNAs regulated by NRF2^a^

miRNA	Chromosome location	Gene targets
miR 193b/365	Chr16, 14397824-14397906 (miR193b) 14403142-14403228 (miR-365)	TTf1 - oncogenic BCL2 - TSG Cyclin D- TSG, uPa
miR-29b	Chr7, 130562218-130562298	Sp-1 MCL-1 - oncogenic TCL1 - oncogenic
miR-181c	Chr19, 13985513-13985622	SIRT1- oncogenic and TSG KRAS - oncogenic TGFβ - TSG TNF - TSG NOTCH - oncogenic and TSG
miR-617	Chr12, 81226312-81226408	N/A
miR-592	Chr7, 126698142-126698238	N/A
miR-1207	Chr8 129061398-129061484	HBEGF
miR-32	Chr9-111808509-111808578	PIK3IP1 - TSG BTG2 - TSG
miR-200c	Chr12, 7072862-7072929	ZEB1, FHOD1, PPM1F, TUBB3-TBK1, BMI1-oncogenic PPP2R1B - TSG
miR-550	Chr7, 30329410-30329506	CPEB4

^a^Location in the chromosome and identified target genes are provided. TSG- tumor suppressor gene (adapted from^[183]^)

## Role of NRF2 in cancer: dysregulation-consequences-drug resistance

Several studies have reported an increased expression of NRF2 in cancers compared to normal cells, with this being one of the chemoprotective roles of NRF2 in cancers^[85,187-192]^. Evidence indicates that a dysregulated NRF2/KEAP1 system, for example KEAP1 mutation^[76,193]^ or NRF2 mutation^[194]^, can be responsible for NRF2 overexpression in cancers leading to enhanced cellular proliferation and chemoresistance^[76,187,193-195]^. NRF2 tends to be overexpressed in cancers when it is freed from KEAP1 anchoring in the cytoplasm at the oxidative state and then translocates to the nucleus, where it heterodimerizes with sMAF and binds to ARE. This, in turn, leads to the expression of cytoprotective and detoxifying genes, such as NQO1 and heme oxygenase-1 (HO-1). This confers protection to cancer cells against ROS-induced apoptosis and DNA damage, thereby enabling cancer cell survival and growth. Nuclear NRF2 expression due to activation of NRF2-ARE signalling may promote tumor progression and drug resistance, and hence NRF2 inhibition could be a strategic path in cancer treatment^[54,58,196]^.

Studies have now focussed on the inhibition of NRF2 to overcome the prolonged or uncontrolled activation of NRF2 in causing tissue damage or cancer progression and chemoresistance. However, the screening, discovery and development of specific, potent, and non-toxic NRF2 inhibitors, including retinoids (e.g., Retinoic Acid, RA and Bexarotene) remains challenging. Potential strategies for developing specific inhibitors include: (1) transcriptional down-regulation of NRF2; (2) increased degradation of NRF2 mRNA for subsequently decreased translation; (3) enhancement of NRF2 degradation, through up-regulation of KEAP1-CUL3 complex, β-TrCP-SCF or HRD1; (4) blocking the translocation of NRF2 to the nucleus leading to antagonising or blocking the dimerization of NRF2 with sMAF proteins; and (5) blocking the NRF2-sMAF DNA-binding domain^[54,58,196]^. It is also worth noting that the Cullin-RING ligases, which are involved in KEAP1 binding and degradation of NRF2, play important roles in human physiology and pathology including cancer^[197,198]^. These molecules also represent potential targets for therapy^[199,200]^.

A review by Namani *et al*.^[54]^ described retinoids as structurally related to vitamin A and other natural and synthetic signalling compounds including retinol, retinal, RA and retinyl esters. They are reported to have an anti-cancer effect because of their proapoptotic and antioxidant activities. Retinoids interact with two different nuclear receptor families, namely retinoic acid receptors (RARs) and retinoid X receptors (RXRs), and these are members of the steroid/thyroid hormone receptor super-family. The RARs themselves contain the three isotypes RARα, RARβ, and RARγ encoded by the *RARA*, *RARB*, and *RARG* genes, and function as ligand-dependent TFs. There are two important isoforms of RARα (α1 and α2) and RARγ (γ1 and γ2) with vital functions; however, RARβ has β1, β2, β3, β4, and β1ʹ isoforms) resultant from differential use of promoters and alternative splicing^[54,58,196]^.

Generally, RARs form heterodimers with RXRs and in the absence of ligand, an RAR/RXR heterodimer can interact with multiple co-repressor proteins such as the nuclear receptor co-repressor and silencing mediator of RA that regulates the transcription of target genes^[54,58,196]^. Also, endogenous ligands such as RAs act as agonists and activate the RAR/RXR heterodimer complex, leading to a reduction in the affinity between the co-repressor and the complex. The coactivator proteins such as steroid receptor coactivators SRC-1, SRC-2, and SRC-3 and proteins that have histone acetyltransferase activity similar to p300-CBP, P300/CBP-associated factor, have general control of amino acid synthesis protein 5-like 2. This will then subsequently interact with high affinity for the RAR/RXR heterodimer, which transactivates the genes targeted by RA through binding to downstream DNA response elements, known as RA response elements^[54,58,196]^.

The nuclear receptors are regulated either in a ligand-dependent or a ligand-independent manner, for example, RXRα physically interacts with NRF2, forms a protein-protein complex and then negatively regulates *ARE* gene expression. Studies have reported that nuclear receptors play dual roles in the aetiology of cancer. For example, PPARγ has been reported to play the role of both tumor promoter and tumor inhibitor in cancers^[54,58,196]^.

The application of siRNA to overcome resistance to chemotherapy and radiotherapy provides a promising therapeutic modality for cancer and other diseases^[201-205]^. A combination of siRNA-mediated gene silencing with natural products has been reported to down-regulate the NRF2-dependent response and partly sensitise MCF-7/TAM cells to tamoxifen in a synergistic manner^[205]^. Another study by Duong *et al*.^[204]^ reported that NRF2-mediated silencing using siRNA reduced the level of aldehyde dehydrogenase 1 family, member A1 and aldehyde dehydrogenase 3 family and member A1; as well as glutamate-cysteine ligase catalytic subunit expression leading to enhanced antiproliferative effects of the chemotherapeutic agent, 5-FU in pancreatic cancer cells.

## NRF2 and its interaction with DNA damage pathways

DNA damage response pathways protect normal cells from environmental damage however these pathways are also major contributors to drug resistance, since they repair the intended damage produced by DNA-targeted cytotoxic drugs. Key DNA damage response pathways include the homologous repair and base excision repair pathways^[206]^ and several modes of co-operative interaction between NRF2 and these pathways have been identified. Mechanisms of interaction include: (1) binding of DNA damage response molecules to NRF2 to stabilise NRF2; (2) co-operation between DNA damage response pathway molecules and NRF2 to enhance gene transcription; and (3) NRF2 regulated transcription of DNA damage response gene expression.

Mutations in the BRCA1 tumor suppressor gene (a component of homologous recombination repair) are associated with increased genomic stability and suggested to account for up to 10% of breast and ovarian cancers^[207]^. BRCA1 regulates NRF2 signalling by at least two mechanisms - it can physically interact with NRF2, thereby promoting its stability and activation^[207]^ and it can bind to NRF2’s promoter and regulate NRF2’s transcription^[208]^. BRCA-1 deficient cells therefore have increased ROS levels as a result of reduced NRF2-mediated antioxidant signalling^[207]^. In an intriguing study using genetically engineered mice, this interaction between NRF2 and BRCA1 has been proposed to explain why BRCA1 deficiency results in an increased incidence of breast or ovarian cancers^[209]^. Since mutation of BRCA1 results in loss of its ability to partner and stabilise NRF2, this would normally lead to cell death from oxidative stress. However, oestrogen, acting via the PI3K/Akt pathway in breast and ovarian cancer cells, can stimulate NRF2 in BRCA1-deficient cells and help sustain these cells which increase their genomic instability eventually leading to malignancy^[210]^.

Molecular co-operation between NRF2 and PARP-1 (involved in base excision repair) has also been demonstrated. While PARP1 does not physically interact with NRF2 or promote NRF2 expression, it has been shown to directly bind to both ARE and small Maf proteins, thereby enhancing NRF2 binding to the ARE and upregulating NRF2 target gene transcription. Hence, PARP1 acts as a transcriptional co-activator indicating a novel function for PARP-1^[211]^.

Analysis of genes regulated by NRF2 has identified multiple genes involved in DNA damage repair pathways. Regulation of these genes by NRF2 is therefore likely to influence resistance. Expression of both Ataxia telangiectasia mutated (ATM) and Ataxia telangiectasia and Rad3 related (ATR) are under NRF2 control^[212]^. The repression of total ATM and ATR protein levels following NRF2 inhibition suggests transcriptional regulation of these kinases by NRF2. NRF2 may directly bind to ATM and ATR promoter regions and repress their expression, or act via indirect means, whereby, it might transcribe another protein, which in turn might regulate ATM and ATR transcription^[212]^. NRF2 has also been associated with the regulation of basal transcription of BRCA1^[213]^. Overexpression of NRF2 increased BRCA1 expression while knockdown of NRF2 attenuated BRCA1 expression. NRF2 was also shown to interact with CBP and p300 to form a transcription complex that bound to the ARE site on the BRCA1 promoter^[213]^. BLAST analysis has been performed on upstream regions of DNA repair genes to identify AREs and it has been demonstrated that many repair genes that are involved in the homologous recombination pathway may be regulated by NRF2^[214]^. These include RAD51D, RAD52 and RAD51C. Other genes that participate in homologous recombination that have ARE sequences in their promoter regions include DMC1, SHFM1, RBBP8 and XRCC3/RAD51C. NRF2 inhibition led to significant reduction in mRNA levels of RAD51^[214]^.

## NRF2 and targeted therapy

Most cancer types have been found to overexpress NRF2 and targeting NRF2 pathway could lead to the identification of a better therapy for NRF2 overexpressing cancers. Inhibition of NRF2 signalling pathway can be achieved by the transcriptional downregulation of NRF2, increased degradation of *NRF2* mRNA or decreased translation, enhancement of NRF2 degradation through upregulation/activation of KEAP1-CUL3, β-TrCP-SCF, or HRD1; blocking the dimerization of NRF2 with small Maf proteins; and blocking the NRF2-sMaf DNA-binding domain^[215]^. In addition to targeted immunotherapy, the use of small molecule kinase inhibitors was also found to be successful in treating various types of cancers. Protein kinases are the most attractive group of drug targets after G-protein-coupled receptors and can be found downstream or upstream of oncogenes or tumor suppressors^[216,217]^. Receptor tyrosine kinases (RTKs) acts as relay points for signalling pathways and are important targets for tyrosine kinase inhibitor (TKI) agents. TKIs, compete with the ATP binding site of the catalytic domain of several tyrosine kinases, and act as small molecules that have a favourable safety profile in disease treatment^[218]^. More than 30 different RTKs have been implicated in cancer and epidermal growth factor receptor (EGFR) system has been reported to be the most prevalent deregulated RTKs which enables them to be chosen as a prototype for drug discovery target^[217,219]^.

**Figure 4:**
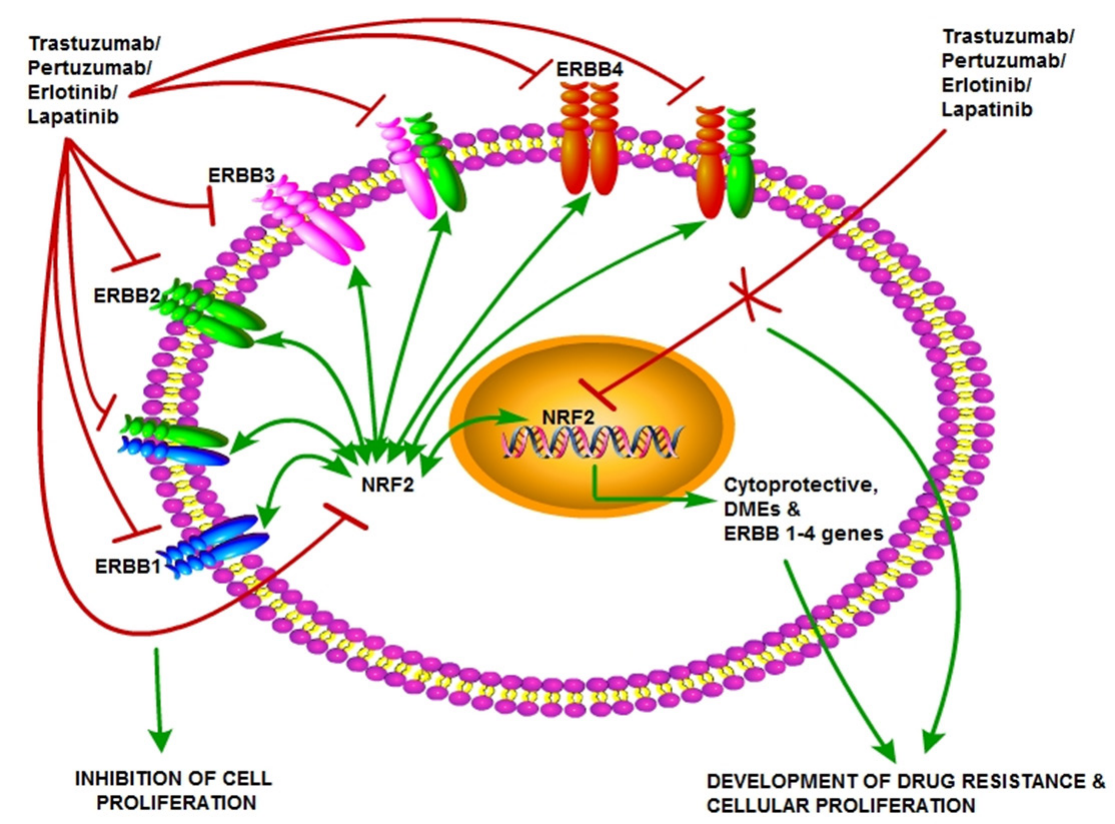
Cooperativity of NRF2 and HER family receptors in cellular proliferation and cancer. The NRF2-ARE pathway cross-talks with the HER family receptor and other signalling pathways. This provides rationale and justification for the use and design of anticancer drugs and/or molecules that target HER family receptors and/or target NRF2 to enhance the action and effectiveness of HER targeting anticancer therapies and delay or overcome resistance. NRF2: nuclear factor E2-related factor 2

The EGFR system is a family of related receptors such as ErbB1 (HER1), ErbB2 (HER2), ErbB3 (HER3), and ErbB4 (HER4) which share ligands and form heterodimers to initiate various signalling events in cell proliferation and survival^[220-223]^. Various studies have shown that NRF2 plays a significant role in action mechanism of many of the targeted therapeutic agents including TKIs. Notably, we have reported that HER receptor targeting immunotherapeutic (Trastuzumab and Pertuzumab) and chemotherapeutic (Erlotinib and Lapatinib) agents act through NRF2 inhibition^[26,27]^. Previously, our group has reported a new mechanism of crosstalk between NRF2 mediated antioxidant response pathway and HER2/HER3 pathway with the use of gene transcriptional reporter assays, pharmacological activation or SiRNA knockdown of NRF2, and HER2/HER3 functional inhibition and activation strategies^[224]^. Inhibition of NRF2 by Pertuzumab and Trastuzumab or their combination leads to disruption of the antioxidant pathway and attenuation of HER2/HER3 signalling^[26]^. Further, we could demonstrate that Erlotinib and Lapatinib could lead to both transcriptional and translational repression of HER1^[27]^ and HER4 (unpublished data) [Figure 4].

**Figure 5:**
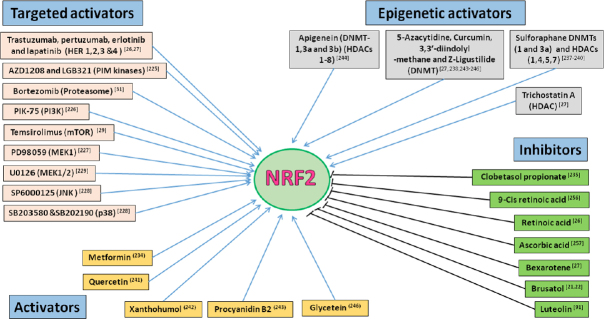
NRF2 modulators with anticancer activity. Small molecule compound or drugs that directly or indirectly modulate NRF2 activity by either activating or inhibiting NRF2 activity and functions. The benefits and risks of modulating NRF2 pathway activity in patients are not fully captured and understood. However, the development of novel NRF2 inhibitors used in combination with existing anticancer drugs could be rational strategy to arrest and mitigate the emergence of chemoresistance to anticancer agents. NRF2: nuclear factor E2-related factor 2

Therapies that target the proteasome are also demonstrated to involve NRF2 modulation [Figure 5]. One proteasome inhibitor, Bortezomib has been shown to induce NRF2 levels, and NRF2 overexpression proteasome maturation protein axis leads to its resistance in multiple myeloma^[31]^. Mechanistic target of rapamycin (mTOR) is a master growth regulator and its inhibition is one of the best options to treat several cancer types. One of the mTOR targeting agent, Temsirolimus has been reported to elicit its effectiveness as an anticancer agent by inhibiting NRF2 in acute myelogenous leukemia stem cells^[29]^. Another family of kinases, the Proviral Integration site for Moloney murine leukemia virus (PIM) kinases are associated with cell growth, differentiation and apoptosis. Overexpression of these kinases has been demonstrated to relate to poor prognosis in various cancers^[30]^.

Small molecule pan-PIM kinase inhibitors such as AZD1208 and LGB321 have been reported to inhibit nuclear accumulation and transcriptional activity of NRF2 which could be responsible for their cytotoxic effect in prostrate and colon cancer cells^[225]^. Further, it has been demonstrated that various other receptor kinases such as PI3K and mitogen-activated protein kinases also exploit NRF2 inhibition as one of their mechanisms of action. PIK-75, a PI3K inhibitor was reported to inhibit NRF2 and augment the sensitivity of gemcitabine in pancreatic cancer cells^[226]^. Several pharmacological inhibitors of MAPK family such as PD98059, MEK1 inhibitor; SB202190 and SB203580, p38 inhibitor; U0126, MEK1/2 inhibitor and SP6000125, JNK inhibitor were reported to be capable of NRF2 inhibition while eliciting their action^[227-230]^.

## Role of NRF2 in the mechanism of action and effectiveness of anticancer drugs

Anticancer drug induced responses can be influenced by TFs like NRF2, which can induce transient or acquired drug resistance. Several mechanisms are proposed to account for the drug resistance phenotype and many of the genes reported to play roles in drug resistance are identified with a functional link with NRF2^[13]^. In anticancer chemotherapy, NRF2 and NRF-dependent genes have been implicated in the cellular resistance to a wide range of anticancer agents (e.g., tamoxifen, Cisplatin, Oxaliplatin, Cisplatin, Doxorubicin, and Etoposide) and cancer types^[18-25]^. Likewise, the NRF2-centred system and signalling pathway is shown to modulate the action and effectiveness of certain receptor targeted therapies^[26-28,224,231,232]^ and potentially promoting cancer resistance to such interventions as Trastuzumab, Pertuzumab, Erlotinib, Lapatinib, imatinib, Gefitinib, Afatinib and Osimertinib. In both anticancer chemotherapy and receptor target therapy, the inhibition of NRF2 and its function seemingly and contextually enhanced drug sensitisation of cancers and/or helped to overcome drug resistance.

Certain drugs conventionally used to treat nonmalignant diseases are currently repurposed to treat cancer, as many of the drugs have been reported to possess potential anticancer actions. Interestingly, some of these drugs have been reported to be NRF2 modulators. Combining these NRF2 modulating repurposed drugs with conventional anticancer chemotherapy and/or receptor target therapeutics has improved the action and effectiveness of these anticancer agents. For example, one of the standard first line therapies for type 2 diabetes mellitus, metformin possesses anti-mitotic, anti-angiogenic and anti-inflammatory activities^[216,233]^. It has been depicted that NRF2 downregulation is involved in metformin mediated reversal of Cisplatin resistance in lung cancer cells^[215]^. Further, high dismal overall survival and breast cancer-specific survival rate has been observed in breast cancer patients with type 2 diabetes mellitus who received metformin with decreased cytoplasmic NRF2 levels^[234]^. Clobetasol propionate, a drug used to treat dermatological diseases has been identified to possess anticancer activity. Choi and colleagues have reported Clobetasol propionate to be a potent NRF2 inhibitor and used it to sensitize lung cancer cells to Rapamycin^[235]^.

Retinoids and rexinoids have been elucidated to be sensitizing chemotherapeutics through NRF2 inhibition. Bexarotene used in the treatment of cutaneous T cell lymphoma is a specific ligand for RXR and has been reported to be an efficacious NRF2 inhibitor. Our previous studies have shown that Bexarotene sensitizes ovarian cancer cells to HER targeted therapeutics such as Erlotinib and Lapatinib through NRF2 inhibition^[27]^. Retinoic acid also has been informed to be improving the sensitivity of ovarian cancer cells to Trastuzumab and Pertuzumab^[26]^ and of breast cancer cells to Cisplatin or Taxol^[236]^ through NRF2 inhibition via a mechanism possibly involving RARs (38/243)^[80,237]^.

## NRF2 and epigenetic modulation

Anticancer agents not only inhibit NRF2 but there are a large group of drugs that activate NRF2. In 2014, Mcmahon *et al*.^[58]^ tested a panel of 152 anticancer agents and found that 10% of the tested drugs were NRF2 inducers. Among them, preclinical targeted therapeutic agents such as insulin like growth factor 1 receptor inhibitor, NVP-AEW541; PIM-1 kinase inhibitor, PIM-1 inhibitor 2; polo like kinase 1 inhibitor, BI 2536 and importantly seven of nine tested HDAC inhibitors were noted to be NRF2 activators^[58]^. This opens up the fact that NRF2 is epigenetically regulated and understanding the mechanism of action of epigenetic modulating anticancer agents in NRF2-ARE pathway is critical for successful cancer treatment. Aberrant activation of NRF2 by epigenetic modulations leads to high expression of cytoprotective proteins thereby decreasing the efficacy of chemotherapy in cancers. Transmission of phenotypic changes from one generation to another with no accompanying alterations in the DNA sequence is known as epigenetics. Epigenetic modulations include the following; DNA methylation/demethylation by DNA methyl transferases (DNMTs), histone modifications by HDACs, and miRNA mediated regulation^[238]^. These epigenetic dysregulations may lead to modifications in the transcription and expression of genes involved in the regulation of cell proliferation and differentiation, cell cycle, and apoptosis^[239-243]^.

Earlier our group demonstrated that the inhibition of HDAC by Trichostatin A and DNMT by 5-azacytidine induced NRF2, HO-1 and transcriptional antioxidant response, and disrupted immunotherapy (Trastuzumab and/or Pertuzumab) dependent repression of NRF2. Epigenetic study of *NRF2* promoter involving CpG methylation profiling confirmed the epigenetic regulation of NRF2 in ovarian cancer cells while receiving HER2 inhibition therapy^[26]^. Another epigenetic modifier Sulforaphane (an HDAC inhibitor) has been reported to activate NRF2 not only by interacting with Keap1 but also by epigenetic mechanisms. Sulforaphane inhibits DNMTs (1 and 3a), and HDACs (1,4,5,7) which reduced CpGs methylation level and increased histone 3 acetylation at the NRF2 promoter^[244-247]^. A study by Kang and colleagues has shown that high NRF2 expression resulting from oxidative stress-induced DNA demethylation promotes 5-FU resistance in colon cancer cells^[248]^. Tri-methylation on K4 of histone H3 by the mixed lineage leukemia (MLL) protein leads to transcriptional activation^[249]^ and the MLL knockdown in colon cancer cells leads to NRF2 and HO-1 down regulation further supporting the epigenetic regulation of NRF2^[248]^. In addition, there are reports on the epigenetic modification (CpG demethylation) of NRF2 by anticancer phytochemicals such as Curcumin, 3,3ʹ-diindolylmethane, Z-Ligustilide, Apigenin, or Tanshinone IIA while eliciting anticancer effect^[245,250-253]^.

## NRF2-feedback and feed forward loops

**Figure 6:**
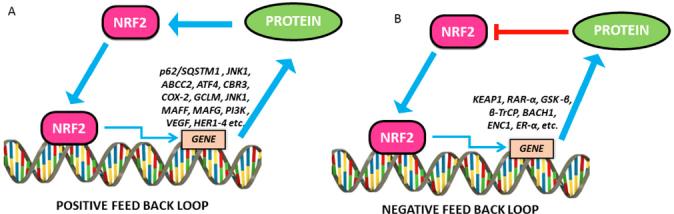
Positive and negative feedback loops in NRF2 signalling. A: The positive feedback regulatory loop of NRF2 and other proteins and signalling pathways that upregulate NRF2 and functions. Showing, in part, the mutual regulatory loop between NRF2 and HER family receptors; B: The negative feedback regulatory loop of NRF2 and other proteins and signalling pathways that repress NRF2 and functions. NRF2: nuclear factor E2-related factor 2

The mechanism behind how cells can achieve a balance between maintaining physiological redox homeostasis and activating the antioxidant system to remove oxidative stress is still unclear. It is proposed that NRF2, one of the master regulators of the antioxidant system through regulating its own degradation to maintain the cellular NRF2 level by an auto regulatory feedback loop will lead to redox homeostasis [Figure 6].

Lee and colleagues have noticed that there was an increase in KEAP1 levels in addition to NRF2 activation when the Hepa-1 cells were exposed to t-BHQ, a known NRF2 activator. KEAP1 promoter and NRF2 knockdown/overexpression studies confirmed that NRF2 could induce KEAP1 promoter activity through binding to an ARE in the reverse strand of proximal promoter. The study further confirmed the transcriptional regulation of KEAP1 by NRF2 whereas KEAP1 controls NRF2 by its degradation^[254]^. A positive feedback loop of NRF2 regulation was NRF2-JNK1 system. JNK1 phosphorylates and induces NRF2 nuclear translocation and whereas NRF2 can transcriptionally induce a battery of stress responsive genes including JNK1^[255,256]^. Another positive feedback loop between p62/SQSTM1 (sequestosome 1) protein, a cargo receptor for autophagic degradation of ubiquitinated targets and NRF2 has been reported. P62 upregulation by oxidative stress is mediated by binding of NRF2 on ARE in the p62 promoter. In addition, it has also been demonstrated that p62 binds directly onto KEAP1 leading to autophagic degradation of KEAP1 and inhibits NRF2 degradation^[257]^.

Another systems-level resource for NRF2 interactome and regulome including 289 protein-protein, 7469 TF-DNA and 85 miRNA interactions has been described. This study identiﬁed that 35 TFs regulated by NRF2 inﬂuence 63 miRNAs that down-regulate NRF2. Among 224 NRF2 interacting proteins 39 of them were found to have regulatory feedback connections with NRF2. In the case of positive feedback, it is signal up regulation whereas in the case of negative feedback it is signal down regulation. NRF2 regulated proteins such as BACH1, ENC1 and ERα has found to be under negative feedback loop whereas the proteins such as ABCC2, ATF4, CBR3, COX-2, GCLM, JNK1, MafF, MafG, PI3K and VEGF were under positive feedback loop^[258]^. Using experimental and systems biology approach, previously our group has elucidated that the basal levels of NRF2 and KEAP1 were cell line speciﬁc and maintained in tight correlation with their growth rates and redox status. Our mathematical model of oxidative stress integrates NRF2-KEAP1 signaling in the cytoplasm and genetic regulation of NRF2-dependent antioxidant enzymes and involves negative feedback between these two control systems^[259]^.

## Conclusion

NRF2 is classically recognised as the master regulator of the cellular antioxidant and cytoprotective defense systems, which confer cellular proliferation, differentiation, migration, organisation and survival of both normal and cancer cells. This role has been paradoxically extended to implicate NRF2 in cellular protection against cancer processes (carcinogenesis) in accelerating and maintaining cancer malignancy following tumor initiation. Generally, the dysregulation and activation of the NRF2 systems are common contributing responsibilities for the pathogenesis of cancers. Further, these NRF2 roles have been recognised or established in several *in vitro* and *in vivo* cancer models, including in pre-clinical and clinical settings. Moreover, the NRF2-dependent defense systems support survival of cancer cells during treatment with chemotherapeutic and target therapeutic agents, as many genes and pathways reported to play roles in anticancer drug resistance seemed to have a regulatory and functional link with NRF2. Collectively, these results imply that upregulation and functional activation of the NRF2 systems are responsible, at least in part, for protection against cancer, for cancer maintenance and progression, and for drug resistance observed during the course of many anticancer therapies. Thus, NRF2 is emerging to be recognised as an oncogene and an as important node and target to modulate and achieve positive outcomes in anticancer therapeutics.

The NRF2 system has therefore appeared to serve as a fundamental redox interconnectivity node and interface between ROS and the regulation of functions in a broad spectrum of cellular physiological and pathological processes including NRF2 cross talk with other signalling and receptor pathways and carcinogenesis. However, it is unclear how cells can achieve a balance between maintaining physiological or pathological redox homeostasis and robustly activate the NRF2 systems to remove exogenous and endogenous ROS to protect cells, or retain some tolerable levels of ROS to confer malignancy or to confer anticancer therapeutic resistance. Several anticancer chemotherapeutic and HER receptor targeting therapies appeared to depend on ROS to effect cytotoxic killing of cancerous cells. Interestingly, some elements of our experimental and modelling work (Khalil *et al*.^[259]^) have indicated that both normal and cancer cells are subjected to oxidative signals that are directed to execute cellular processes such as proliferation, and perhaps cell death, up to a critical threshold, which is defined by an S-type regulation curve. It is conceivable that further developments and future refinements to this NRF2 systems and centred model could lead to a tool to evaluate, predict, tailor, manipulate and manage ROS and to inform physiological or pathological redox homeostasis, cellular behavior state and form, as well as therapeutic strategies like anticancer therapies. Furthermore, it is increasingly clear that NRF2 and its function can be modulated, pharmacologically or genetically, to enhance the action and effectiveness of anticancer chemotherapeutic and/or receptor targeting therapeutics. This has presented the possibility and potentiality of investigating and identifying novel modulators of NRF2 from existing clinical drugs through repositioning or repurposing, which can be directed in combination therapy to augment the action and effectiveness of certain anticancer therapeutic agents, as well as to overcome anticancer therapeutic resistance. Overall, there is unequivocal emerging role of NRF2 in the mechanism of action and resistance to many anticancer therapies and NRF2 is an important candidate target for the design and development of anticancer therapies.

## Declarations

### Copyright

© The Author(s) 2019.
